# Language-concordant automated telephone queries to assess medication adherence in a diverse population: a cross-sectional analysis of convergent validity with pharmacy claims

**DOI:** 10.1186/s12913-018-3071-4

**Published:** 2018-04-06

**Authors:** Neda Ratanawongsa, Judy Quan, Margaret A. Handley, Urmimala Sarkar, Dean Schillinger

**Affiliations:** 1 General Internal Medicine and UCSF Center for Vulnerable Populations at San Francisco General Hospital and Trauma Center, University of California, 1001 Potrero Avenue, Box 1364, San Francisco, CA 94143 USA; 2 Department of Epidemiology and Biostatistics, Division of Preventive Medicine and Public Health, University of California, 1001 Potrero Avenue, Box 1364, San Francisco, CA 94143 USA

**Keywords:** Diabetes, Health information technology, Medication adherence, Safety net clinics, Medicaid, Managed care, Limited health literacy, Limited English proficiency, Health disparities

## Abstract

**Background:**

Clinicians have difficulty accurately assessing medication non-adherence within chronic disease care settings. Health information technology (HIT) could offer novel tools to assess medication adherence in diverse populations outside of usual health care settings. In a multilingual urban safety net population, we examined the validity of assessing adherence using automated telephone self-management (ATSM) queries, when compared with non-adherence using continuous medication gap (CMG) on pharmacy claims. We hypothesized that patients reporting greater days of missed pills to ATSM queries would have higher rates of non-adherence as measured by CMG, and that ATSM adherence assessments would perform as well as structured interview assessments.

**Methods:**

As part of an ATSM-facilitated diabetes self-management program, low-income health plan members typed numeric responses to rotating weekly ATSM queries: “In the last 7 days, how many days did you MISS taking your …” diabetes, blood pressure, or cholesterol pill. Research assistants asked similar questions in computer-assisted structured telephone interviews. We measured continuous medication gap (CMG) by claims over 12 preceding months. To evaluate convergent validity, we compared rates of optimal adherence (CMG ≤ 20%) across respondents reporting 0, 1, and ≥ 2 missed pill days on ATSM and on structured interview.

**Results:**

Among 210 participants, 46% had limited health literacy, 57% spoke Cantonese, and 19% Spanish. ATSM respondents reported ≥1 missed day for diabetes (33%), blood pressure (19%), and cholesterol (36%) pills. Interview respondents reported ≥1 missed day for diabetes (28%), blood pressure (21%), and cholesterol (26%) pills. Optimal adherence rates by CMG were lower among ATSM respondents reporting more missed days for blood pressure (*p* = 0.02) and cholesterol (*p* < 0.01); by interview, differences were significant for cholesterol (*p* = 0.01).

**Conclusions:**

Language-concordant ATSM demonstrated modest potential for assessing adherence. Studies should evaluate HIT assessments of medication beliefs and concerns in diverse populations.

**Trial registration:**

NCT00683020, registered May 21, 2008.

**Electronic supplementary material:**

The online version of this article (10.1186/s12913-018-3071-4) contains supplementary material, which is available to authorized users.

## Background

Suboptimal adherence to hypoglycemic, anti-hypertensive, and lipid-lowering medications contributes to suboptimal cardiometabolic control and poor clinical outcomes [1–4]. Patients with limited health literacy (LHL) and limited English proficiency (LEP) experience communication barriers that may increase their risk for suboptimal adherence and contribute to known disparities in diabetes care and health outcomes [5–15].

Medication adherence can be enhanced by improving patient-centered prescribing and intervening upon barriers to intentional and unintentional non-adherence [16, 17]. However, this improvement process requires that clinicians and care teams elicit patients’ goals, beliefs and concerns to identify motivations and reduce barriers to taking medications [16, 18–20]. Unfortunately, clinicians are often unable to identify non-adherence in their patients [2, 3, 21–28]. Moreover, clinicians face time constraints and competing demands that reduce their ability to engage their patients effectively during visits, particularly in resource-challenged safety net settings that serve vulnerable patients [29–31].

Health information technology (HIT) offers innovative ways to engage linguistically diverse populations outside of usual medical care settings [32–35]. The Institute of Medicine highlighted automated telephone self-management (ATSM) as an innovative HIT strategy to enhance health outcomes for patients with chronic disease and LHL [36]. We previously demonstrated that culturally and linguistically tailored diabetes ATSM is a cost-effective strategy to improve self-care and functional outcomes with high acceptability and engagement in a safety net population [37–40].

Our aim in this study was to evaluate the accuracy of ATSM in assessing chronic diabetes medication adherence within a vulnerable safety net population. Systematic reviews have highlighted the challenges of assessing medication adherence, with particular concern about social desirability bias with self-reported measures [41]. Thus, it is important to evaluate the performance of HIT tools for assessing adherence, compared to that of more objective measures. Validating adherence assessments against cardiometabolic control, such as hemoglobin A1c and blood pressure, may yield inconsistent results because of many factors that affect these outcomes [41]. Prescription refill data offers one common standard against which to assess the convergent validity of self-reported adherence. Continuous medication gap (CMG) is an objective measure of adherence that uses pharmacy dispensings to measure gaps in patients’ available supply of medications [42, 43]. In a recent a cross-sectional study of English-, Spanish-, and Cantonese-speaking urban health plan members with diabetes, 63% had a calculable CMG, and suboptimal adherence by CMG occurred more frequently among members with poor cardiometabolic control than among members with optimal control (28% vs. 12%, *p* = 0.02) [40].

Using CMG as a standard, we conducted this study to investigate the validity of language-concordant ATSM queries to assess patient-reported medication adherence in a diverse low-income population with diabetes. We hypothesized that patients reporting greater days of missed pills to ATSM queries would have higher rates of non-adherence as measured by CMG. As a comparison to a standard clinical and research method for assessing adherence, we also investigated the relationship between missed pills reported during a computer-assisted structured interview and CMG, hypothesizing that ATSM would perform as well as an interview.

## Methods

This cross-sectional analysis uses data from the SMARTSteps Program (Pasos Positivos / 明智進步計劃), a quasi-experimental study of a self-management HIT intervention for San Francisco Health Plan (SFHP) members with diabetes (April 2009 – March 2011) [44, 45]. For this analysis, we included all SMARTSteps participants regardless of their originally assigned intervention arm. We previously published the full description of the SMARTSteps protocol, including the recruitment and randomization flowchart and data collection methods [44, 45].

### Study setting

SFHP (http://www.sfhp.org/) is a non-profit, government-sponsored managed care plan for low-income San Francisco residents. SFHP administers both Medicaid and “Healthy Workers,” an insurance program for in-home support service providers for elderly or disabled people in San Francisco. Members of these plans comprise a large proportion of patients served by the San Francisco Department of Public Health (SFDPH) healthcare system.

SMARTSteps eligibility criteria included: English-, Cantonese-, or Spanish-speaking adults (age ≥ 18) with diabetes and ≥ 1 primary care visit in the preceding 24 months to one of four SFDPH clinics. Members who were pregnant, lacked a touch-tone phone, reported plans to leave the region, or were unable to provide verbal consent were ineligible. We identified diabetes patients through the SFDPH diabetes registry or those with evidence of SFHP claims related to diabetes, followed by confirmation in the SFDPH electronic health record (clinician-documented diagnosis of diabetes, fasting glucose ≥126 mg/dl, or hemoglobin A1c ≥ 7%) [46]. We excluded SFHP members who denied having diabetes during recruitment calls.

### Ethics, consent and permissions

The Committee on Human Research at the University of California, San Francisco (10–04689 and 13–10,752) approved verbal consent for the collection of interview data by UCSF, which they deemed minimal risk [44, 45]. All program recruitment was conducted by SFHP by phone, and so a separate visit to obtain written consent at UCSF or the primary care clinics risked reducing participation and increasing selection bias.

### Data sources

SFHP conducted all recruitment into the SMARTSteps program through mailed post cards and scripted outreach calls. SFHP enrollment workers confirmed eligibility by phone, offered $25 gift card incentives to participate in the program, and assessed willingness to complete evaluation interviews administered by UCSF bilingual research assistants.

After obtaining verbal consent, bilingual research assistants conducted structured, computer-assisted telephone interviews within 2 weeks of participant enrollment (see Additional file 1 “SMARTSteps Baseline Questionnaire”). The instruments – translated in Spanish and Cantonese and then back-translated into English – asked participants to report educational attainment, employment status, and annual household income. Research assistants also screened participants for English proficiency and health literacy with items validated for this population [47–49]. Participants received a $50 gift card.

UCSF researchers had permission from SFHP and SFDPH, respectively, to collect SFHP administrative data (age, gender, race/ethnicity, language, financial class / insurance type, and pharmacy claims) and SFDPH clinical registry data (hemoglobin A1c, systolic blood pressure, diastolic blood pressure, and low-density lipoprotein) obtained through routine care.

### Patient-reported medication adherence assessments

Self-reported adherence to cardiometabolic medications was assessed in two ways: automated telephone self-management queries and patient interview by a research assistant. The wording of the assessment questions for both methods – derived from the Summary of Diabetes Self-Care Activities measure – has evidence of predictive validity for health outcomes in chronic medical conditions [50, 51].

**Automated telephone self-management (ATSM) queries** (see Additional files 2 and 3: “SMARTSteps WEEK 5” and “SMARTSteps WEEK 6”): Tailored for literacy, language, and culture with extensive patient input [37], the ATSM system provided 8–12 min weekly calls for 27 weeks in English, Cantonese, or Spanish. Calls asked participants rotating sets of queries about diabetes medication adherence and self-care, psychosocial issues, and access to preventive services. Based on the responses entered on the touchtone keypad, participants heard different health education messages containing narratives. “Out-of-range” responses triggered callbacks within 3 days from a language-concordant SFHP lay health coaches, who engaged in collaborative goal-setting to form patient-centered action plans. Health coaches – supervised by an SFHP registered nurse care manager – documented their phone encounter content in the SFHP care management database system and transmitted the content to primary care clinics by email, fax, and phone for actionable concerns.

Each of the 27 weeks included a medication adherence question, rotating weekly to focus on a particular therapeutic indication important to risk reduction in diabetes [1–4]: “In the last 7 days, how many days did you MISS taking your …”

- “… diabetes medications, even just one pill or shot?”
- “… blood pressure pills, even just one pill?”
- “… cholesterol medications, even just one pill?”

Participants typed their response (0 to 7) on a telephone keypad. Patients’ responses were based on their understanding of the reasons for taking their medications. All participants answered the same query in a given week, regardless of whether they were prescribed any medication or more than one medication for a given indication.

To enhance disclosure, all questions were preceded by a normalizing statement: [41] “For most people, taking all of their medications every day can be hard. Now we want to ask you about the medications you are taking and how often you take them.”

For this analysis, we included the first completed ATSM response for each therapeutic indication, hypothesizing that the health coaching intervention could have affected responses over time. In sensitivity analyses, to explore potential change in the performance of ATSM during the 27-week intervention, we found that results for the last completed ATSM response were similar to the findings from the first completed ATSM response.

**Patient interview questions:** During the baseline SMARTSteps interview, bilingual research assistants asked all participants who reported having been prescribed or asked by a member of participants’ diabetes health care team to take a diabetes, blood pressure, or cholesterol pill: “In the last 7 days, how many days did you MISS even one …”

- “… diabetes pill?”
- “… blood pressure pill?”
- “… cholesterol pill?”

Participants could choose a response between 0 and 7 days. Patients’ responses were based on their understanding of the reasons for taking their medications, and patients were asked the same questions regardless of whether they were prescribed more than one medication for a given indication. To enhance disclosure, questions were preceded with a normalizing statement [41]: “Many people miss taking their medication sometimes, so it’s okay if you tell me you don’t always take all of your medications.”

### Continuous medication gap (CMG)

CMG is a well-established measure of secondary adherence for ongoing medications using pharmacy data [42, 43]. The validity of the CMG requires that patients obtain all prescriptions within the insurance system from which claims are drawn and have continuous prescription benefits throughout a defined observation period [42, 43, 52]. CMG is calculated if a patient fills a medication at least 2 times during the capture period. This implies that the patient completed at least one “refill interval,” the time between two fills. CMG estimates the proportion of days without sufficient medication supply within each refill interval and then sums those for all refill intervals within the measurement period.

In this analysis, we measured CMG for SMARTSteps participants with active pharmacy benefits in the 12 months preceding the medication adherence assessments (ATSM query and patient interview separately). CMG was calculable if the participant had claims for the relevant therapeutic indication (at least 1 oral diabetes, hypertension, or hyperlipidemia medication) and at least two fills during the 12-month CMG measurement period. CMG can be inaccurate if it doesn’t account for medication stockpiling, and so we used a modified approach with a time-forward algorithm so that extra pills remaining would not negate medication supply gaps during previous refill intervals but would be included as part of the future medication supply [52].

CMG was not calculable for dually eligible Medicaid/Medicare beneficiaries, whose alternative pharmacy benefit coverage prohibits complete ascertainment of pharmacy claims. CMG is also not calculable for insulin because pharmacy utilization data does not provide insulin fixed days supply based on prescribed dosing [3, 53]. Although questions about insulin adherence were included in the ATSM queries and patient interview, we excluded these from this analysis.

For each subject, we calculated CMG separately by indication (i.e., CMG for all oral diabetes medications, CMG for all anti-hypertensive medications, and CMG for all hyperlipidemia medications). Using a previously validated classification, we considered patients as having *optimal adherence* when medications were available for 80% or more of the time (CMG ≤ 20%) and *suboptimal adherence* when patients lacked a medication supply > 20% of the observation time (CMG > 20%) [4, 42, 43].

### Analysis

For each patient-reported adherence assessment method, separately for each therapeutic indication, we categorized responses into three groups based on the distribution of participant responses: 0 days of missed pill, 1 day of missed pill, or 2–7 days of missed pill.

For participants with responses in each of these categories, we first determined whether CMG was calculable, since less adherent patients have fewer fills and are less likely to have a calculable CMG.

To evaluate the convergent validity of each patient-reported adherence assessment method for each therapeutic indication, we used Fisher’s exact tests to compare the prevalence of optimal adherence (CMG ≤ 20%) vs. suboptimal adherence (CMG > 20%) across the 3 categories of participant responses: 0 vs. 1 vs. 2–7 days of missed pills.

To investigate differences in suboptimal adherence disclosure between assessment methods, we explored the proportion of participants who disclosed non-adherence via ATSM query, patient interview, or both. Comparisons were made by therapeutic indication and limited to first completed ATSM adherence responses occurring within 30 days of the patient interview, to maximize sample size while minimizing potential variation in actual adherence behaviors between measurements.

## Results

Among 362 eligible SFHP members randomized to participate in SMARTSteps, 210 completed a baseline interview, had pharmacy benefits within the 12 months before the adherence assessment, and had sufficient fills of at least one cardiometabolic medication. Table 1 describes sociodemographic and medical characteristics of these participants. Patients averaged 56 years in age, and 75% were women. Sixty-four percent were Asian / Pacific Islander, 12% were Latino, and only 24% had their assessments in English (57% in Cantonese and 19% in Spanish). Just under half of members (46%) had limited health literacy, and 63% reported annual household incomes of less than $20,000. The average duration of diabetes was 7.1 years (SD 5.5), with 26% of members having hemoglobin A1c > 8.0% and 21% receiving insulin.

**Table 1:** Baseline sociodemographic and medical characteristics of health plan members receiving a language-concordant automated telephone self-management / health coaching diabetes intervention (*N* = 210)

Characteristic	
Age in years, mean (SD)	56.0 (6.8)
Women, n (%)	157 (74.8)
Race/ethnicity, n (%)	
Latino	44 (20.1)
Black / African-American	12 (5.7)
Asian / Pacific Islander	135 (64.3)
White / Caucasian	14 (6.7)
Multi-Ethnic / Other	5 (2.4)
Born outside the U.S., n (%)	23 (11.0)
Language, n (%)	
Cantonese-speaking	120 (57.1)
Spanish-speaking	39 (18.6)
Language-concordant primary care provider	55 (34.6)
Educational attainment, n (%)	
8th grade education or less	90 (42.9)
Some high school	21 (10.0)
High school graduate or GED	49 (23.3)
College graduate or above	50 (23.8)
Limited health literacy, n (%)	97 (46.4)
Employment status, n (%)	
Employed full-time	51 (24.3)
Part-time	98 (46.7)
Unemployed	24 (11.4)
Disabled	14 (6.7)
Homemaker / Retired / Other	23 (11.0)
Annual household income, n (%)	
≤ $20,000	124 (62.6)
$20,001–30,000	38 (19.2)
> $30,000	36 (18.2)
Insurance type, n (%)	
Medicaid	44 (21.0)
Uninsured/Commercial	2 (1.0)
Healthy Worker/Healthy San Francisco	164 (78.1)
Years diagnosed with diabetes, mean (SD)	7.1 (5.5)
Insulin treatment, n (%)	43 (20.5)
Hemoglobin A1c > 8.0%, n (%)	51 (25.5)
Hemoglobin A1c, mean (SD)	7.7 (1.5)
Systolic blood pressure, mean mm Hg (SD)	126.2 (16.3)
Low-density lipoprotein, mean mg/dL (SD)	87.3 (26.8)

Table 2 shows the rates of optimal adherence (CMG ≤ 20%), separately across therapeutic indications for those participating in the ATSM calls and the interview. The prevalence of optimal adherence among respondents varied by indication: 83% for diabetic pills, 91% for blood pressure pills, and 85–87% for cholesterol pills.

**Table 2:** Mean continuous medication gap (CMG) and prevalence of optimal medication adherence (CMG ≤ 20%) among health plan members receiving automated telephone self-management / health coaching diabetes intervention

	First Completed ATSM			Baseline Interview		
Medication	Number	Mean CMG (SD)	Optimal Adherence	Number	Mean CMG (SD)	Optimal Adherence
Diabetic pill	170	0.08 (0.13)	83.5%	178	0.08 (0.14)	82.6%
Blood pressure pill	113	0.06 (0.12)	91.1%	125	0.06 (0.11)	91.4%
Cholesterol pill	115	0.09 (0.14)	85.2%	130	0.08 (0.12)	86.9%

**Fig. 1:**
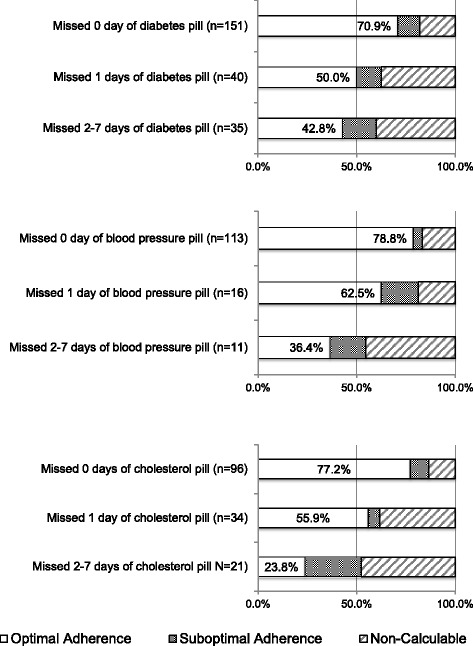
Proportions of automated telephone self-management (ATSM) query respondents who had optimal adherence, suboptimal adherence, or non-calculable continuous medication gap. * *p*-value is for the comparison between optimal and suboptimal adherence

Among ATSM respondents, 75 respondents (33.2%) for diabetes, 19 (19.3%) for blood pressure, and 55 (36.4%) for cholesterol reported at least 1 missed pill day. Figure 1 shows the proportions of ATSM query respondents who had optimal adherence, suboptimal adherence, or non-calculable continuous medication gap for each therapeutic indication (diabetes pill *n* = 226, blood pressure pill *n* = 140, cholesterol pill *n* = 151). The charts show that prevalence rates of optimal adherence were lower for ATSM respondents who reported more missed days, with significant differences across response categories for blood pressure (*p* = 0.02) and cholesterol (*p* < 0.01) but not for diabetes (*p* = 0.19).

**Fig. 2:**
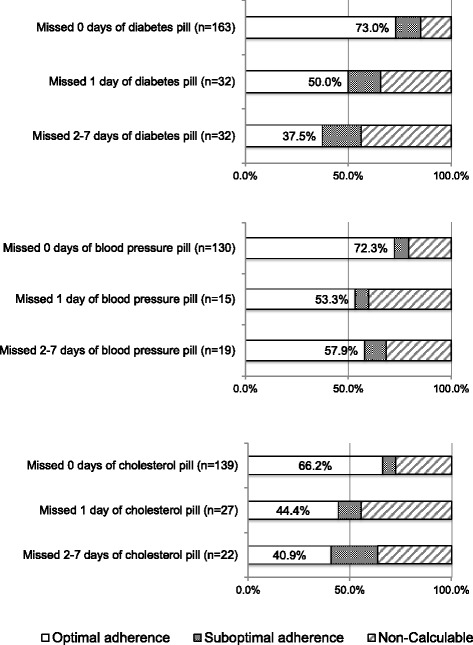
Proportions of interview respondents who had optimal adherence, suboptimal adherence, or non-calculable continuous medication gap. * *p*-value is for the comparison between optimal and suboptimal adherence

Among interview respondents, 64 respondents (28.2%) for diabetes, 34 (20.7%) for blood pressure, and 49 (26.1%) for cholesterol reported at least 1 missed pill day. Figure 2 shows the proportions of interview respondents who had optimal adherence, suboptimal adherence, or non-calculable continuous medication gap for each therapeutic indication (diabetes pill *n* = 227, blood pressure pill *n* = 164, cholesterol pill *n* = 188). The adherence prevalence rates across interview response categories were significant different for cholesterol (*p* = 0.01), but not for diabetes (*p* = 0.11) or blood pressure (0.49).

We analyzed a small sample of patients who answered both questions on the ATSM system and the interview within a 30-day timeframe (Table 3). No consistent pattern suggested missed days were disclosed more often by ATSM vs. interview.

**Table 3:** Concordance of reports of missed medication days between ATSM and interview responses among health plan members receiving automated telephone self-management / health coaching diabetes intervention

	Diabetes pill (*n* = 84)	Blood pressure pill (*n* = 51)	Cholesterol pill (*n* = 55)
Concordant	71.4%	72.5%	54.6%
Higher disclosure on interview	11.9%	21.6%	14.5%
Higher disclosure on ATSM	16.7%	5.9%	30.9%

## Discussion

In a diverse low-income LHL and LEP population with diabetes, ATSM assessment of self-reported adherence performed as well as, if not better than, interview by a research analyst. This suggests that language-concordant ATSM offers a potential methodology to assess medication adherence asynchronously as an adjunct to usual clinical care, as a health surveillance tool to assist in population health outreach, and as a research tool when working with linguistically diverse safety net populations.

Patient self-report is the most common method used in research and clinical care for assessing medication non-adherence. The National Institutes of Health Adherence Network reviewed current research on the validity of self-report measures, concluding that they tend to overestimate adherence compared with other measures and have weak sensitivity, but high specificity [41]. However, the review panel still endorsed self-report measures as the “most practical method” for assessing adherence in clinical care due to their “low-cost, noninvasiveness, minimal patient burden, ease of administration, and flexibility in timing and mode of administration” [41].

ATSM offers several advantages for assessing self-reported adherence. First, ATSM is available outside the context of usual medical care at a time and location convenient to patients, with high engagement rates across patients with different chronic medical conditions [45, 54]. This can facilitate panel management by care management teams, targeted outreach to high risk patients, and more effective and efficient future visits with prescribing clinicians. Second, it allows the systematic delivery of content tailored to the language and literacy of a safety net population [37, 38]. A large survey of diverse public hospital patients found that LHL patients and Spanish-speaking Hispanics particularly preferred telephonic delivery of self-management support, compared with group visits or internet-based programs [55]. Third, like computer-assisted structured interviews, ATSM may help minimize social desirability bias and facilitate disclosure of suboptimal adherence [41]. Overall, the SMARTSteps ATSM queries met several of this NIH Adherence Network panel’s recommendations for improving the validity of self-report measures, including the use of: previously validated questions, technology rather than face-to-face collection to reduced social desirability bias, introductory normalizing statements, and specific recall periods [41].

However, ATSM and other HIT-facilitated adherence assessment tools remain imperfect and insufficient without complementary strategies for assessing adherence [56, 57]. In our study, some patients with suboptimal adherence by CMG disclosed missed medication days on ATSM, some disclosed during an interview, and some did not disclose at all. Some differences may relate to differences in the adherence assessment time frames, with ATSM and interview using a 7-day recall and CMG using a 1-year capture period. In addition, participants’ responses to ATSM and interview questions were based on their own understanding of the reasons for taking medications, rather than a pharmacologic indication, and so mismatch (e.g., a patient considering their “benazepril” to be a “diabetes pill”) may partially explain these differences. However, some differences may relate to comfort disclosing non-adherence via different modalities. This comfort may be affected by a patient’s expectations for the response that may follow disclosure and a patient’s trust in their providers, health care teams, and health system to use this disclosure to improve their treatment plan [7, 25]. In the SMARTSteps intervention, ATSM was not solely used for adherence assessment but embedded within a larger intervention to provide self-management support through recorded narratives and health coaching follow-up calls. As such, we designed the health coaching scripts to capture and address participants’ broader concerns and beliefs about the necessity of medications [16, 44]. Thus, HIT-facilitated adherence assessments are only one tool among the necessary resources for engaging patients about their medication-taking behaviors and beliefs.

The limitations of the study should be noted. First, the timeframes for assessing adherence in the self-report questions (7 days) differed from the capture period for CMG (1 year). A short recall period for eliciting self-reported adherence is recommended by adherence experts because it facilitates disclosure and minimizes recall bias, particularly among older patients with memory difficulties [41]. However, health care professionals are usually interested in patients’ medication-taking behaviors over a longer period than 7 days, and so our analysis remains useful for evaluating these ATSM queries’ utility for clinical practice. Second, CMG was not calculable among some participants, particularly among those with higher numbers of self-reported missed days. Our statistical comparison of optimal and suboptimal adherence is likely conservative, since most participants without a calculable CMG would have had suboptimal adherence because they lacked adequate numbers of pharmacy dispensings to calculate CMG. Third, we lacked prescribing data by participants’ clinicians, which could have led to overestimates of adherence (for those with primary non-adherence who never got medications filled) or underestimates of adherence (for those whose medications were discontinued by prescribing providers during the CMG capture period). Fourth, we did not specifically assess patients’ perceptions of the acceptability of ATSM adherence assessment, although we have previously found high engagement with ATSM overall in this population [37–40]. Fifth, our findings are affected by selection bias and may have limited generalizability to other populations. Finally, although we did not pursue subgroup analyses due to concerns about power to detect meaningful differences, future studies should investigate how HIT-facilitated, language-concordant adherence assessments perform across race/ethnicity, educational level, health literacy, or English proficiency.

## Conclusions

ATSM demonstrated modest potential for assessing self-reported medication adherence in a diverse, multilingual, low-income population with diabetes. Future studies should explore the potential for HIT platforms to evaluate the multiple dimensions of medication adherence, including the goals, values, beliefs, and concerns of diverse populations.

## Additional files

Additional file 1: SMARTSteps Baseline Questionnaire. This document contains sections of the baseline patient interview instrument relevant to analysis in the article “Language-Concordant Automated Telephone Queries to Assess Medication Adherence in a Diverse Population: A Cross-Sectional Analysis of Convergent Validity with Pharmacy Claims”. (PDF 163 kb)

Additional file 2: SMARTSteps WEEK 5. This document contains the English-language scripted queries delivered through an automated telephone self-management program to patients with diabetes. These queries include examples of the questions about medication adherence for diabetes and cholesterol pills, as analyzed in the article “Language-Concordant Automated Telephone Queries to Assess Medication Adherence in a Diverse Population: A Cross-Sectional Analysis of Convergent Validity with Pharmacy Claims”. (PDF 105 kb)

Additional file 3: SMARTSteps WEEK 6. This document contains the English-language scripted queries delivered through an automated telephone self-management program to patients with diabetes. These queries include examples of the questions about medication adherence for diabetes and blood pressure pills, as analyzed in the article “Language-Concordant Automated Telephone Queries to Assess Medication Adherence in a Diverse Population: A Cross-Sectional Analysis of Convergent Validity with Pharmacy Claims”. (PDF 114 kb)

## Abbreviations

ATSM: Automated telephone self-management
CMG: Continuous medication gap
HIT: Health information technology
LEP: Limited English proficiency
LHL: Limited health literacy
SFDPH: San Francisco Department of Public Health
SFHP: San Francisco Health Plan
SMARTSteps: Self-Management Automated and Real-Time Telephonic Support
UCSF: University of California, San Francisco

## References

[1] Kerr EA, Zikmund-Fisher BJ, Klamerus ML, Subramanian U, Hogan MM, Hofer TP (2008). The role of clinical uncertainty in treatment decisions for diabetic patients with uncontrolled blood pressure. Ann Intern Med.

[2] Heisler M, Hogan MM, Hofer TP, Schmittdiel JA, Pladevall M, Kerr EA (2008). When more is not better: treatment intensification among hypertensive patients with poor medication adherence. Circulation.

[3] Schmittdiel JA, Uratsu CS, Karter AJ, Heisler M, Subramanian U, Mangione CM, Selby JV (2008). Why don't diabetes patients achieve recommended risk factor targets? Poor adherence versus lack of treatment intensification. J Gen Intern Med.

[4] Karter AJ, Parker MM, Moffet HH, Ahmed AT, Schmittdiel JA, Selby JV (2009). New prescription medication gaps: a comprehensive measure of adherence to new prescriptions. Health Serv Res.

[5] Bailey SC, Brega AG, Crutchfield TM, Elasy T, Herr H, Kaphingst K, Karter AJ, Moreland-Russell S, Osborn CY, Pignone M, Rothman R, Schillinger D (2014). Update on health literacy and diabetes. Diabetes Educ.

[6] McNaughton CD, Jacobson TA, Kripalani S (2014). Low literacy is associated with uncontrolled blood pressure in primary care patients with hypertension and heart disease. Patient Educ Couns.

[7] White RO, Osborn CY, Gebretsadik T, Kripalani S, Rothman RL (2013). Health literacy, physician trust, and diabetes-related self-care activities in Hispanics with limited resources. J Health Care Poor Underserved.

[8] Osborn CY, Cavanaugh K, Wallston KA, Kripalani S, Elasy TA, Rothman RL, White RO (2011). Health literacy explains racial disparities in diabetes medication adherence. J Health Commun.

[9] Gazmararian JA, Kripalani S, Miller MJ, Echt KV, Ren J, Rask K (2006). Factors associated with medication refill adherence in cardiovascular-related diseases: a focus on health literacy. J Gen Intern Med.

[10] 2014 National Healthcare Quality & Disparities Report (2015). Agency for Healthcare Research and Quality, Rockville, MD.

[11] Fernandez A, Schillinger D, Warton EM, Adler N, Moffet HH, Schenker Y, Salgado MV, Ahmed A, Karter AJ (2011). Language barriers, physician-patient language concordance, and glycemic control among insured Latinos with diabetes: the diabetes study of northern California (DISTANCE). J Gen Intern Med.

[12] Sarkar U, Handley MA, Gupta R, Tang A, Murphy E, Seligman HK, Shojania KG, Schillinger D (2010). What happens between visits? Adverse and potential adverse events among a low-income, urban, ambulatory population with diabetes. Qual Saf Health Care.

[13] Schillinger D, Barton LR, Karter AJ, Wang F, Adler N (2006). Does literacy mediate the relationship between education and health outcomes? A study of a low-income population with diabetes. Public Health Rep.

[14] Davis TC, Wolf MS, Bass PF, Thompson JA, Tilson HH, Neuberger M, Parker RM (2006). Literacy and misunderstanding prescription drug labels. Ann Intern Med.

[15] Schillinger D, Bindman A, Wang F, Stewart A, Piette J (2004). Functional health literacy and the quality of physician-patient communication among diabetes patients. Patient Educ Couns.

[16] Horne R, Chapman SC, Parham R, Freemantle N, Forbes A, Cooper V (2013). Understanding patients’ adherence-related beliefs about medicines prescribed for long-term conditions: a meta-analytic review of the necessity-concerns framework. PLoS One.

[17] Capoccia K, Odegard PS, Letassy N (2016). Medication adherence with diabetes medication: a systematic review of the literature. Diabetes Educ.

[18] Heisler M, Hofer TP, Schmittdiel JA, Selby JV, Klamerus ML, Bosworth HB, Bermann M, Kerr EA (2012). Improving blood pressure control through a clinical pharmacist outreach program in patients with diabetes mellitus in 2 high-performing health systems: the adherence and intensification of medications cluster randomized, controlled pragmatic trial. Circulation.

[19] Krahn M, Naglie G (2008). The next step in guideline development: incorporating patient preferences. JAMA.

[20] Montori VM, Fernandez-Balsells M (2009). Glycemic control in type 2 diabetes: time for an evidence-based about-face?. Ann Intern Med.

[21] Meddings J, Kerr EA, Heisler M, Hofer TP (2012). Physician assessments of medication adherence and decisions to intensify medications for patients with uncontrolled blood pressure: still no better than a coin toss. BMC Health Serv Res.

[22] Grant RW, Pabon-Nau L, Ross KM, Youatt EJ, Pandiscio JC, Park ER (2011). Diabetes oral medication initiation and intensification: patient views compared with current treatment guidelines. Diabetes Educ.

[23] Montori VM, Gafni A, Charles C (2006). A shared treatment decision-making approach between patients with chronic conditions and their clinicians: the case of diabetes. Health Expect.

[24] Elwyn G, Hutchings H, Edwards A, Rapport F, Wensing M, Cheung WY, Grol R (2005). The OPTION scale: measuring the extent that clinicians involve patients in decision-making tasks. Health Expect.

[25] Kraetschmer N, Sharpe N, Urowitz S, Deber RB (2004). How does trust affect patient preferences for participation in decision-making?. Health Expect.

[26] McCaffery KJ, Smith SK, Wolf M (2010). The challenge of shared decision making among patients with lower literacy: a framework for research and development. Med Decis Mak.

[27] Makoul G, Clayman ML (2006). An integrative model of shared decision making in medical encounters. Patient Educ Couns.

[28] Saba GW, Wong ST, Schillinger D, Fernandez A, Somkin CP, Wilson CC, Grumbach K (2006). Shared decision making and the experience of partnership in primary care. Ann Fam Med.

[29] Linzer M, Bitton A, Tu SP, Plews-Ogan M, Horowitz KR, Schwartz MD, Poplau S, Paranjape A, Landry M, Babbott S, Collins T, Caudill TS, Prasad A, Adolphe A, Kern DE, Aung K, Bensching K, Fairfield K, Association of Chiefs and Leaders in General Internal Medicine (ACLGIM) Writing Group* (2015). The end of the 15-20 minute primary care visit. J Gen Intern Med.

[30] Linzer M, Manwell LB, Williams ES, Bobula JA, Brown RL, Varkey AB, Man B, McMurray JE, Maguire A, Horner-Ibler B, Schwartz MD, MEMO (Minimizing Error, Maximizing Outcome) Investigators (2009). Working conditions in primary care: physician reactions and care quality. Ann Intern Med.

[31] Varkey AB, Manwell LB, Williams ES, Ibrahim SA, Brown RL, Bobula JA, Horner-Ibler BA, Schwartz MD, Konrad TR, Wiltshire JC, Linzer M, MEMO Investigators (2009). Separate and unequal: clinics where minority and nonminority patients receive primary care. Arch Intern Med.

[32] Thakkar J, Kurup R, Laba TL, Santo K, Thiagalingam A, Rodgers A, Woodward M, Redfern J, Chow CK (2016). Mobile telephone text messaging for medication adherence in chronic disease: a meta-analysis. JAMA Intern Med.

[33] Aikens JE, Trivedi R, Aron DC, Piette JD (2015). Integrating support persons into diabetes telemonitoring to improve self-management and medication adherence. J Gen Intern Med.

[34] Rana AI, van den Berg JJ, Lamy E, Beckwith CG (2016). Using a mobile health intervention to support HIV treatment adherence and retention among patients at risk for disengaging with care. AIDS Patient Care STDs.

[35] Horvath T, Azman H, Kennedy GE, Rutherford GW (2012). Mobile phone text messaging for promoting adherence to antiretroviral therapy in patients with HIV infection. Cochrane Database Syst Rev.

[36] IOM (Institute of Medicine) (2009). Health literacy, eHealth, and communication: putting the consumer first: workshop summary.

[37] Schillinger D, Hammer H, Wang F, Palacios J, McLean I, Tang A, Youmans S, Handley M (2008). Seeing in 3-D: examining the reach of diabetes self-management support strategies in a public health care system. Health Educ Behav.

[38] Schillinger D, Handley M, Wang F, Hammer H (2009). Effects of self-management support on structure, process, and outcomes among vulnerable patients with diabetes: a three-arm practical clinical trial. Diabetes Care.

[39] Handley MA, Shumway M, Schillinger D (2008). Cost-effectiveness of automated telephone self-management support with nurse care management among patients with diabetes. Ann Fam Med.

[40] Ratanawongsa N, Karter AJ, Quan J, Parker MM, Handley M, Sarkar U, Schmittdiel JA, Schillinger D (2015). Reach and validity of an objective medication adherence measure among safety net health plan members with diabetes: a cross-sectional study. J Manag Care Spec Pharm.

[41] Stirratt MJ, Dunbar-Jacob J, Crane HM, Simoni JM, Czajkowski S, Hilliard ME, Aikens JE, Hunter CM, Velligan DI, Huntley K, Ogedegbe G, Rand CS, Schron E, Nilsen WJ (2015). Self-report measures of medication adherence behavior: recommendations on optimal use. Transl Behav Med.

[42] Steiner JF, Prochazka AV (1997). The assessment of refill compliance using pharmacy records: methods, validity, and applications. J Clin Epidemiol.

[43] Steiner JF, Koepsell TD, Fihn SD, Inui TS (1988). A general method of compliance assessment using centralized pharmacy records. Description and validation. Med Care.

[44] Ratanawongsa N, Handley MA, Quan J, Sarkar U, Pfeifer K, Soria C, Schillinger D (2012). Quasi-experimental trial of diabetes self-management automated and real-time telephonic support (SMARTSteps) in a Medicaid managed care plan: study protocol. BMC Health Serv Res.

[45] Ratanawongsa N, Handley MA, Sarkar U, Quan J, Pfeifer K, Soria C, Schillinger D (2014). Diabetes health information technology innovation to improve quality of life for health plan members in urban safety net. J Ambul Care Manage.

[46] American Diabetes Association (2009). Standards of medical care in diabetes--2009. Diabetes Care.

[47] Wilson E, Chen AH, Grumbach K, Wang F, Fernandez A (2005). Effects of limited English proficiency and physician language on health care comprehension. J Gen Intern Med.

[48] Sarkar U, Schillinger D, Lopez A, Sudore R (2011). Validation of self-reported health literacy questions among diverse English and Spanish-speaking populations. J Gen Intern Med.

[49] Chew LD, Griffin JM, Partin MR, Noorbaloochi S, Grill JP, Snyder A, Bradley KA, Nugent SM, Baines AD, Vanryn M (2008). Validation of screening questions for limited health literacy in a large VA outpatient population. J Gen Intern Med.

[50] Toobert DJ, Hampson SE, Glasgow RE (2000). The summary of diabetes self-care activities measure: results from 7 studies and a revised scale. Diabetes Care.

[51] Wu JR, DeWalt DA, Baker DW, Schillinger D, Ruo B, Bibbins-Domingo K, Macabasco-O'Connell A, Holmes GM, Broucksou KA, Erman B, Hawk V, Cene CW, Jones CD, Pignone M (2014). A single-item self-report medication adherence question predicts hospitalisation and death in patients with heart failure. J Clin Nurs.

[52] Bryson CL, Au DH, Young B, McDonell MB, Fihn SD (2007). A refill adherence algorithm for multiple short intervals to estimate refill compliance (ReComp). Med Care.

[53] Ratanawongsa N, Karter AJ, Parker MM, Lyles CR, Heisler M, Moffet HH, Adler N, Warton EM, Schillinger D. Communication and medication refill adherence: the diabetes study of northern California. JAMA Intern Med. 2013;173(3):210–8. 10.1001/jamainternmed.2013.1216 PMC3609434 23277199

[54] Piette JD, Rosland AM, Marinec NS, Striplin D, Bernstein SJ, Silveira MJ (2013). Engagement with automated patient monitoring and self-management support calls: experience with a thousand chronically ill patients. Med Care.

[55] Sarkar U, Piette JD, Gonzales R, Lessler D, Chew LD, Reilly B, Johnson J, Brunt M, Huang J, Regenstein M, Schillinger D (2008). Preferences for self-management support: findings from a survey of diabetes patients in safety-net health systems. Patient Educ Couns.

[56] Bender BG, Bartlett SJ, Rand CS, Turner C, Wamboldt FS, Zhang L (2007). Impact of interview mode on accuracy of child and parent report of adherence with asthma-controller medication. Pediatrics.

[57] Hettema JE, Hosseinbor S, Ingersoll KS (2012). Feasibility and reliability of interactive voice response assessment of HIV medication adherence: research and clinical implications. HIV Clin Trials.

