# Supplementary material for: Language-concordant automated telephone queries to assess medication adherence in a diverse population: a cross-sectional analysis of convergent validity with pharmacy claims
Source: BMC Health Serv Res. 2018 Apr 6;18:254. doi: 10.1186/s12913-018-3071-4 (PMC5889590; doi:10.1186/s12913-018-3071-4)
Supplement: Supplementary file 1 — SMARTSteps Baseline Questionnaire. This document contains sections of the baseline patient interview instrument relevant to analysis in the article “Language-Concordant Automated Telephone Queries to Assess Medication Adherence in a Diverse Population: A Cross-Sectional Analysis of Convergent Validity with Pharmacy Claims”. (PDF 163 kb) [file 12913_2018_3071_MOESM1_ESM.pdf]

SMARTSteps Baseline Questionnaire

Sections relevant to analysis in article "Language-Concordant Automated Telephone Queries to Assess Medication Adherence in a Diverse Population: A Cross-Sectional Analysis of Convergent Validity with Pharmacy Claims"

DEMOGRAPHICS

Now I'd like to ask about some basic information about you.

How many years have you had diabetes? **[If <1 yr, use decimal, e.g. 6 mos = 0.5 yr. Otherwise, round to the nearest integer.]**

________YEARS

Were you born in the U.S.?

1 YES

0 NO ⟶ In total, how many years have you lived in the U.S.? _______ YEARS

What ethnic group do you **most** identify with? **[Check one only]**

1 HISPANIC OR LATINO
2 NON-HISPANIC OR NON-LATINO

What race or group do you **most** identify with? **[Check one only]**

1 ASIAN OR PACIFIC ISLANDER
2 BLACK OR AFRICAN AMERICAN
3 WHITE OR CAUCASIAN
4 NATIVE AMERICAN
5 LATINO (A) OR HISPANIC
6 **[Don't read]** MULTI-ETHNIC [eg. 1& 3]
7 **[Don't read]** OTHER _____________
8 **[Don't read]** REFUSED

Are you currently married or in a long-term relationship?

1 YES
0 NO

What is the highest level of school that you have completed?

1 NEVER WENT TO SCHOOL
2 BETWEEN 1ST AND 5TH GRADE
3 BETWEEN 6TH AND 8TH GRADE
4 SOME HIGH SCHOOL
5 HIGH SCHOOL GRADUATE OR "GED" DEGREE
6 SOME COLLEGE OR TECHNICAL SCHOOL
7 COLLEGE GRADUATE

8 GRADUATE DEGREE

Which of the following best describes your current working status?

1 WORKING FULL-TIME (≥ 35 HR) IF YES: _________HRS/ WEEK
2 PART-TIME (<35 HR) IF YES: _________HRS/ WEEK
3 HOMEMAKER
4 UNEMPLOYED
5 DISABLED
6 RETIRED
7 **[Don't read]** OTHER _______

Which of the following categories best describes your total annual household income? Please remember, this survey is confidential.

1 LESS THAN $5,000 PER YEAR
2 BETWEEN $5,001 AND $10,000 PER YEAR
3 BETWEEN $10,001 AND $20,000 PER YEAR
4 BETWEEN $20,001 AND $30,000 PER YEAR
5 MORE THAN $30,000 PER YEAR
8 **[Don't read]** REFUSED
9 **[Don't read]** DON'T KNOW

Medications

Now I will ask you some questions about your medications. Some questions may ask about your **diabetes health care team. This team includes people from the San Francisco Health Plan and from your clinic (doctors, nurses, nutritionists, dieticians, pharmacists, diabetes educators, telephone nurses and counselors).** Many people miss taking their medication sometimes, so it's okay if you tell me you don't always take all of your medications.

In the last 6 months…

Have you been prescribed or asked by a member of your diabetes health care team to take insulin?

1 YES, AND I **CURRENTLY** TAKE INSULIN
2 YES, BUT I **DO NOT** TAKE INSULIN
0 NO, I WAS **NEVER** PRESCRIBED OR ASKED TO TAKE INSULIN
9 **[Don't read]** DON'T KNOW

[IF BOTTOM 3, SKIP TO NEXT SECTION]

When you are feeling GOOD, do you sometimes STOP taking your insulin shots?

□1 Yes: You stop because…[Please choose **ALL** that apply]
1 insulin makes you feel sick (side effects)
2 you don't think insulin is helpful
3 you think insulin might cause additional problems
4 you might gain weight
5 insulin makes you feel low/ blue/ suffer
6 insulin costs a lot
7 you already take too many medications
8 other

□0 No: You always take them.

When you are feeling SICK, do you sometimes STOP taking your insulin shots?

□1 Yes: You stop because…[Please choose **ALL** that apply]
1 they make you feel sick (side effects)
2 you don't think they are helpful
3 you think they might cause additional problems
4 you might gain weight
5 they make you feel low/ blue/ suffer
6 they cost a lot
7 you already take too many medications
8 other

□0 No: You always take them.

Thinking about the **last 7 days**, how many days did you MISS even one insulin shot?
[Read options]

0 0 DAYS [I DID NOT MISS ANY SHOTS]
1 1 DAY

2 2 DAYS
3 3 DAYS
4 4 DAYS
5 5 DAYS
6 6 DAYS
7 7 DAYS

If your diabetes health care team were to recommend that you INCREASE the dose of your insulin, would you agree to do so?

☐1 Yes

☐0 No: Is this because… [Please choose **ALL** that apply]

1 they make you feel sick (side effects)
2 you don't think they will be helpful
3 you think they might cause additional problems
4 you might gain weight
5 they make you feel low/ blue/ suffer
6 they cost a lot
7 you already take too many medications
8 other

If <u>NOT</u> currently using Insulin:

If your diabetes health care team recommended you START insulin, would you start?

1 YES
0 NO: Is this because… [Please choose **ALL** that apply]

1it would make you feel sick (side effects)
2 you don't think it will be helpful
3 you think it might cause additional problems
4 you might gain weight
5 it might make you feel low/ blue/ suffer
6 it might cost a lot
7 you already take too many medications
8 other
9 **[Don't read]** DON'T KNOW

In the <u>last 6 months</u>…

Have you been prescribed or asked by a member of your diabetes health care team to take diabetes pills?

1 YES, AND I **CURRENTLY** TAKE DIABETES PILLS
2 YES, BUT I **DO NOT** TAKE DIABETES PILLS
0 NO , I WAS **NEVER** PRESCRIBED OR ASKED TO TAKE DIABETES PILLS
9 **[Don't read]** DON'T KNOW

[IF BOTTOM 3, SKIP TO NEXT SECTION]

When you are feeling GOOD, do you sometimes STOP taking your diabetes pills?

☐1 Yes: You stop because…[Please choose **ALL** that apply]

1 they make you feel sick (side effects)

2 you don't think they are helpful
3 you think they might cause additional problems
4 you might gain weight
5 they make you feel low/ blue/ suffer
6 they cost a lot
7 you already take too many medications
8 other

☐0 No: You always take them.

When you are feeling SICK, do you sometimes STOP taking your diabetes pills?
☐1 Yes: You stop because…[Please choose **ALL** that apply]
1 they make you feel sick (side effects)
2 you don't think they are helpful
3 you think they might cause additional problems
4 you might gain weight
5 they make you feel low/ blue/ suffer
6 they cost a lot
7 you already take too many medications
8 other

☐0 No: You always take them.

In the **last 7 days**, how many days did you MISS even one diabetes pill?
[Read options]

0 0 DAYS
1 1 DAY
2 2 DAYS
3 3 DAYS
4 4 DAYS
5 5 DAYS
6 6 DAYS
7 7 DAYS

If your diabetes health care team were to recommend that you INCREASE the dose of your diabetes pills, would you agree to do so?
☐1 Yes

☐0 No: Is this because…[Please choose **ALL** that apply]
1 they make you feel sick (side effects)
2 you don't think they are helpful
3 you think they might cause additional problems
4 you might gain weight
5 they make you feel low/ blue/ suffer
6 they cost a lot
7 you already take too many medications
8 other

If your diabetes health care team were to recommend that you take a NEW diabetes pill, would you take it?

1 YES

0 NO: Is this because… [Please choose **ALL** that apply]

- 1 it would make you feel sick (side effects)
- 2 you don't think it will be helpful
- 3 you think it might cause additional problems
- 4 you might gain weight
- 5 it might make you feel low/ blue/ suffer
- 6 it might cost a lot
- 7 you already take too many medications
- 8 other

If NOT currently using Diabetes pills:

If your diabetes health care team recommended you START diabetes pills, would you start?

1 YES

0 NO: Is this because… [Please choose **ALL** that apply]

- 1 it would make you feel sick (side effects)
- 2 you don't think it will be helpful
- 3 you think it might cause additional problems
- 4 you might gain weight
- 5 it might make you feel low/ blue/ suffer
- 6 it might cost a lot
- 7 you already take too many medications
- 8 other
- 9 **[Don't read]** DON'T KNOW

In the last 6 months…

Have you been prescribed or asked by a member of your diabetes health care team to take blood pressure pills?

1 YES AND I **CURRENTLY** TAKE BLOOD PRESSURE PILLS

2 YES, BUT I **DO NOT** TAKE BLOOD PRESSURE PILLS

0 NO, I WAS **NEVER** PRESCRIBED OR ASKED TO TAKE BLOOD PRESSURE PILLS

9 **[Don't read]** DON'T KNOW

[IF BOTTOM 3, SKIP TO NEXT SECTION]

When you are feeling GOOD, do you sometimes STOP taking your blood pressure pills?

☐1 Yes: You stop because…[Please choose **ALL** that apply]

- 1 they make you feel sick (side effects)
- 2 you don't think they are helpful
- 3 you think they might cause additional problems
- 4 they make you feel low/ blue/ suffer
- 5 they cost a lot
- 6 you already take too many medications
- 8 other

☐0 No: You always take them.

When you are feeling SICK, do you sometimes STOP taking your blood pressure pills?

☐1 Yes: You stop because…[Please choose **ALL** that apply]

1 they make you feel sick (side effects)
2 you don't think they are helpful
3 you think they might cause additional problems
4 they make you feel low/ blue/ suffer
5 they cost a lot
6 you already take too many medications
8 other

☐0 No: You always take them.

In the **<u>last 7 days</u>**, how many days did you MISS even one blood pressure pill? **[Read options]**

0 0 DAYS
1 1 DAY
2 2 DAYS
3 3 DAYS
4 4 DAYS
5 5 DAYS
6 6 DAYS
7 7 DAYS

If your diabetes health care team were to recommend that you INCREASE the dose of your blood pressure pills, would you agree to do so?

☐1 Yes

☐0 No: Is this because… [Please choose **ALL** that apply]

1 they make you feel sick (side effects)?
2 you don't think they will be helpful
3 you think they might cause additional problems
4 they make you feel low/ blue/ suffer
5 they cost a lot
6 you already take too many medications
8 other

If your diabetes health care team were to recommend that you take a NEW medication for blood pressure, would you take it?

1 YES
0 NO: Is this because… [Please choose **ALL** that apply]

1 it would make you feel sick (side effects)
2 you don't think it will be helpful
3 you think it might cause additional problems
4 it might make you feel low/ blue/ suffer
5 it might cost a lot

6 you already take too many medications
8 other

If NOT currently using Blood Pressure pills:
80. If your diabetes health care team recommended you START taking blood pressure pills, would you start?

1 YES
0 NO: Is this because… [Please choose **ALL** that apply]
 1 they would make you feel sick (side effects)
 2 you don't think they will be helpful
 3 you think they might cause additional problems
 4 they might make you feel low/ blue/ suffer
 5 they might cost a lot
 6 you already take too many medications
 8 other
 9 **[Don't read]** DON'T KNOW

In the last 6 months…
Have you been prescribed or asked by a member of your diabetes health care team to take pills to lower your cholesterol?

1 YES, AND I **CURRENTLY** TAKE CHOLESTEROL PILLS
2 YES, BUT I **DO NOT** TAKE CHOLESTEROL PILLS
0 NO , I WAS **NEVER** PRESCRIBED OR ASKED TO TAKE CHOLESTEROL PILLS
9 **[Don't read]** DON'T KNOW

[IF BOTTOM 3, SKIP TO NEXT SECTION]

When you are feeling GOOD, do you sometimes STOP taking your cholesterol pills?
□1 Yes: You stop because…[Please choose **ALL** that apply]
 1 they make you feel sick (side effects)
 2 you don't think they are helpful
 3 you think they might cause additional problems
 4 they make you feel low/ blue/ suffer
 5 they cost a lot
 6 you already take too many medications
 8 other

□0 No: You always take them.

When you are feeling SICK, do you sometimes STOP taking your cholesterol pills?
□1 Yes: You stop because…[Please choose **ALL** that apply]
 1 they make you feel sick (side effects)
 2 you don't think they are helpful
 3 you think they might cause additional problems
 4 they make you feel low/ blue/ suffer
 5 they cost a lot

6 you already take too many medications
8 other

□0 No: You always take them.

In the **last 7 days**, how many days did you MISS even one cholesterol pill?
[Read options]

0 0 DAYS
1 1 DAY
2 2 DAYS
3 3 DAYS
4 4 DAYS
5 5 DAYS
6 6 DAYS
7 7 DAYS

If your diabetes health care team were to recommend that you INCREASE the dose of your cholesterol pills, would you agree to do so?
□1 Yes

□0 No: Is this because… [Please choose **ALL** that apply]
1 they make you feel sick (side effects)
2 you don't think they will be helpful
3 you think they might cause additional problems
4 they make you feel low/ blue/ suffer
5 they cost a lot
6 you already take too many medications
8 other

If your diabetes health care team were to recommend that you take a NEW medication for cholesterol, would you take it?
1 YES
0 NO: Is this because… [Please choose **ALL** that apply]
1it would make you feel sick (side effects)
2 you don't think it will be helpful
3 you think it might cause additional problems
4 it might make you feel low/ blue/ suffer
5 it might cost a lot
6 you already take too many medications
8 other

If NOT currently using Cholesterol pills:
87. If your diabetes health care team recommended you START taking cholesterol pills, would you start?
1 YES
0 NO: Is this because… [Please choose **ALL** that apply]

1 it would make you feel sick (side effects)
2 you don't think it will be helpful
3 you think it might cause additional problems
4 it might make you feel low/ blue/ suffer
5 it might cost a lot
6 you already take too many medications
8 other
9 **[Don't read]** DON'T KNOW

In the last 6 months…
Have you been prescribed or asked by a member of your diabetes health care team to take aspirin?

1 YES, AND I **CURRENTLY** TAKE ASPIRIN
2 YES, BUT I **DO NOT** TAKE ASPIRIN
0 NO , I WAS **NEVER** PRESCRIBED OR ASKED TO TAKE ASPIRIN
9 **[Don't read]** DON'T KNOW

[IF BOTTOM 3, SKIP TO NEXT SECTION]

In the **last 7 days**, how many days did you MISS even one aspirin pill?
[Read options]

0 0 DAYS
1 1 DAY
2 2 DAYS
3 3 DAYS
4 4 DAYS
5 5 DAYS
6 6 DAYS
7 7 DAYS

HEALTH LITERACY

QHELP, QLEARN, & QFORM

Now we'd like to ask you some questions about your experience with written or printed information that you might get from your diabetes team, or at the clinic, hospital or pharmacy. Please try to answer the questions as best as you can.

How often do you have someone like a family member, friend, hospital or clinic worker or caregiver, help you read clinic or hospital materials?

5 ALWAYS
4 OFTEN
3 SOMETIMES
2 RARELY
1 NEVER

How often do you have problems learning about your medical condition because of difficulty understanding written information?

5 ALWAYS
4 OFTEN
3 SOMETIMES
2 RARELY
1 NEVER

How confident are you filling out forms by yourself?

5 EXTREMELLY CONFIDENT
4 QUITE A BIT CONFIDENT
3 SOMEWHAT CONFIDENT
2 A LITTLE CONFIDENT
1 NOT AT ALL CONFIDENT
